# Chronic kidney disease in sugarcane workers in Cameroon: a cross-sectional study

**DOI:** 10.1186/s12882-017-0798-9

**Published:** 2018-01-15

**Authors:** Martin E. Ekiti, Jean-Berthelot Zambo, Felix K. Assah, Valirie N. Agbor, Krystel Kekay, Gloria Ashuntantang

**Affiliations:** 1 Faculty of Medicine and Biomedical Sciences, University of Yaoundé I, P.O. Box 1364, Yaounde, Cameroon; 2 Cameroon Sugar Company (SOSUCAM), Mbandjock, Cameroon; 3 Ibal sub-Divisional Hospital, Oku, Northwest Region, Bamenda, Cameroon; 4 Occupational Health Service, National Refining Company (SONARA), P.O. Box 365, Limbe, Cameroon

**Keywords:** Chronic kidney disease, Sugarcane plantation workers, Sub-Saharan Africa, Cameroon

## Abstract

**Background:**

Agricultural workers especially in sugarcane plantations have a high risk of chronic kidney disease (CKD). Little is known about CKD among sugarcane plantation workers in Cameroon. This study sought to evaluate the prevalence and identify factors associated with CKD in sugarcane plantation workers in Cameroon.

**Methods:**

We conducted an analytic cross-sectional study including 204 adult workers at the sugarcane plantation complex in Mbandjock, Cameroon; over 500 m above sea level. Chronic kidney disease (proteinuria as estimated by urine dipstick analysis and/or estimated glomerular filtration rate < 60 ml/min/1.73 m^2^ persistent after 3 months) was the outcome of interest. Those with abnormal results were seen again after 3 months to confirm the diagnosis. We evaluated the association between CKD and participant age, sex, contract-type, duration of employment, socio-economic status, workspace, exposure to agrochemicals, heavy metals and heat, selected risk factors and co-morbid conditions**.**

**Results:**

The overall prevalence of CKD was 3.4%. The factory workers were the most affected (7%), compared to the field (2.4%) and office workers (0%). 2.9% of the participants had persistent proteinuria, mild in every case, and 0.5% of them had an estimated glomerular filtration rate < 60 ml/min/1.73 m^2^. Age ≥ 40 years was an independent predictor of CKD.

**Conclusion:**

The prevalence of CKD among employees of the Mbandjock sugarcane plantation is low, probably reflecting the preventive measures against heat stress and dehydration in place.

## Background

Chronic kidney disease (CKD) is a major public health problem due to its high global prevalence (13.4%) [1], and adverse outcomes such as end-stage renal disease (ESRD), cardiovascular disease (CVD), and premature death [2]. Diabetes mellitus, hypertension and chronic glomerulonephritis constitute the major causes of CKD worldwide [2]. The overall prevalence of CKD in Cameroon is unknown. However, prevalence rates of 10.9% and 14.1% have been reported in an urban and rural setting of the Western Region of Cameroon, respectively [3].

Agricultural workers are considered a high risk population for CKD in several parts of the world. The prevalence of CKD in plantation communities in Central America, Egypt, India and Sri Lanka has been reported to exceed the global prevalence of CKD in the general population [4, 5]. In El Salvador, Vela et al. showed a CKD prevalence of 50.2% in two farming communities [6]. Several plantations especially sugarcane plantations have been identified as risk environments. Torres et al. found a GFR < 60 ml/min/1.73m^2^ prevalence rate of 17% among male sugarcane plantation workers in Nicaragua [7], while Peraza et al. reported a similar prevalence rate of 18% among male sugarcane plantation workers in El Salvador [8].

In Cameroon, agriculture is a major occupation as the country hosts one of the three largest sugarcane farming regions in SSA [9]. To the best of our knowledge, there is no data on the prevalence of CKD among sugarcane plantation workers in SSA in general, and Cameroon in particular. With the aim of contributing to the knowledge in this area in SSA, we conducted this study to evaluate the prevalence and associated factors of CKD among sugarcane plantation workers in the Mbandjock sugarcane plantation in Cameroon.

## Methods

### Study design, setting and sampling

We conducted an analytical cross-sectional study from November 2015 to May 2016 at the sugarcane plantation (SOSUCAM) complex in Mbandjock, which is a town about 100 km north-east of Yaoundé, the capital city of Cameroon. Consenting adults having worked for the company for at least 6 months were eligible. Interns, pregnant and post-partum women and participants with insufficient laboratory data were excluded.

For our study, we used the following formula for cross-sectional studies to calculate the sample size:

$$ n=\frac{{Z_{1-\alpha /2}}^2p\left(1-p\right)}{d^2} $$

Where:

n = Sample size.

p = Expected proportion in population.

d = Absolute error or precision.

Z_1-α/2_ = Standard normal variate for significance (1.96 if type I error is limited to 5%).

**Fig. 1:**
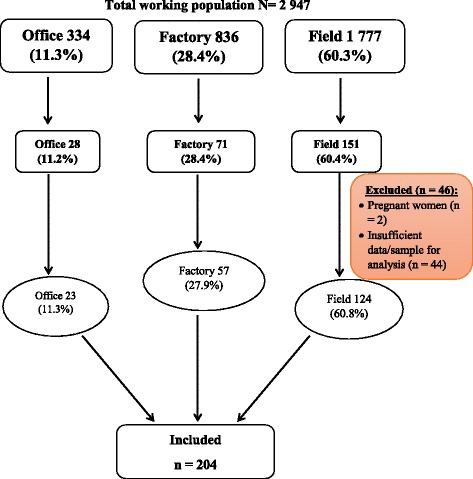
Participant recruitment flowchart – To make our sample population representative of the general working population we randomly selected pre-numbered workers such that the proportions, with respect to workspace, were respected at every stage. In the first stage, we selected 250 participants. After excluding 46, we retained 204 participants, ensuring that the proportions remained approximately the same

The expected proportion (p) of CKD patients in a rural general population in Cameroon, based on studies carried out by Kaze et al. is 14.1% [3]. We tolerated an absolute error (d) of 5%. Using the above formula, the minimum sample size calculated was 187 study participants. We used a stratified random sampling method to select 204 participants with workspace (office, factory or field) being the stratification variable such that the representation of participants according to workspace was similar to that in the general working population (Fig. 1).

### Measurements

Socio-demographic data and relevant elements of the past history such as contract-type (permanent, temporary, seasonal), duration of employment, workspace (office, factory, field), exposure (to agrochemicals, heavy metals and heat), personal and family history of risk factors (hypertension, diabetes, CKD, chronic use of herbal medicines, chronic use of non-steroidal anti-inflammatory drugs, alcohol use, tobacco use) were noted using pre-established questionnaires. Anthropometric parameters, blood pressure and body mass index measurement was done using standard methods in line with the WHO STEPS survey methods. Blood and urine samples were collected in the mornings before any job activity. After disinfecting each participant’s hands, they were handed a clean dry urine cup and instructed on how to collect midstream urine. A random morning urine sample was collected for dipstick urinalysis (Mission® Expert of ACON® Laboratories, USA). Venous blood was collected for serum creatinine assay which was done in the biochemistry laboratory of the SOSUCAM medico-surgical center with a semi-automatic chemistry analyser (Mindray® model No BA-88A) applying the kinetic Jaffe reaction within 12 h. Capillary blood was used to measure blood sugar level.

After 3 months, urine dipsticks and serum creatinine assay were repeated for participants with abnormal results at baseline. GFR was estimated using the CKD-EPI eq. [10].

### Definitions

CKD was defined as the persistence of proteinuria and/or GFR <60 ml/min over a period of 3 months. Urine abnormalities were defined as the presence of ≥1+ of protein, blood or leucocytes on dipsticks. Proteinuria was categorized as mild (1+, 30-100 mg/dl), moderate (2+, 100-500 mg/dl) or heavy (3+, >500 mg/dl). Factory workers were mainly those involved in preparing the material for the planting of the crop, those exploiting the harvested crop and those maintaining the factory. Field workers were those doing the actual planting, maintenance and harvesting of the crop. All backpack sprayers of pesticides, herbicides and fertilizers, and those who mixed and stored them were considered exposed to agrochemicals. We considered welders who work on lead pipes and laboratory technicians who use lead acetate to be exposed to heavy metals. All field workers and factory workers in the boiler section were considered exposed to heat. Participants who used NSAIDs and herbal medicines at least once every month were considered to be chronic users. Seasonal workers were those who were hired on a temporary basis during a particular season, either the harvest or planting season, whose contracts were suspended for the 3 month period between both seasons. Participants on anti-diabetic agents or having a fasting or random blood sugar level > 126 or 200 mg/dl respectively were considered diabetic while those on antihypertensive medication or with a blood pressure > 140/90 mmHg were considered hypertensive.

Socioeconomic status (SES) was evaluated using a questionnaire adapted from the Cameroon Demographic and Health Survey’s household questionnaire [11]. Low SES described people living in lack who cook with wood and coal, live in mud houses with no basic electrical appliances. They earn very little and drink water of dubious quality. Middle SES described people who possess certain goods which are not particularly essential to survival for example a television, a radio, a portable gas cooker and a telephone. Generally they drink potable water but of public use. Their average monthly income is about 150,000 FCFA ($250). High SES described people who have vehicles (motor bikes or cars), have individual water sources at home and are generally autonomous in terms of electricity supply. Their average monthly income is about 250,000 FCFA ($430).

### Statistical analysis

Data collected was analyzed using SPSS version 20. Continuous variables were presented as means ± SD or median with interquartile range (IQR) where necessary. Categorical variables were presented as frequencies and percentages. A two-sided Chi-squared, or Fisher’s exact test where applicable, was used to compare qualitative variables. The Student t-test was used to compare means between two groups. A one-way analysis of variance (ANOVA), or the Kruskal-Wallis test where appropriate, was used to compare means or medians between the groups divided according to workspace. A multivariate logistic regression model was used to determine factors independently associated with CKD while controlling for confounders. The threshold of significance was set at 0.05 with 95% confidence intervals.

### Ethical considerations

The study was approved by the Cameroon National Ethics Committee for Human Health Research. During an introductory meeting, the study was presented to each participant. They were each given an information sheet according to their language of choice (English or French) to read and those who had difficulties had the information explained to them by the principal investigator (EM). They each gave their written informed consent by signing the consent form.

## Results

### General characteristics of the study population

There were 2947 employed workers at the time of the study who met our eligibility criteria; 334 office workers, 836 factory workers and 1777 field workers. After sampling, we approached 252 workers to participate in the study two of whom refused to participate, giving an acceptance rate of 99.2%. We then excluded 2 pregnant women and 44 participants for insufficient data for analysis leaving us with 204 participants (Fig. 1).

Of the 204 participants, 23 (11.3%) were office employees, while 57 (27.9%) and 124 (60.8%) were factory and field workers respectively. The mean age of our participants was 39 ± 10 years and the median duration of employment was 10 years [IQR = 9.75]. The mean age and duration of employment were similar across all three workgroups. There were significantly more men (75%) and seasonal workers (73.5%) in all workspaces (Table 1).

**Table 1:** Characteristics of sugarcane plantation workers according to workspace in Mbandjock, Centre Region, Cameroon, 2016

Variable	Total, n (%) *N* = 204	Office, n (%) *N* = 23	Factory, n (%) *N* = 57	Field, n (%) *N* = 124	*p*-value
Sex					<0.001
Male	153 (75.0)	17 (73.9)	55 (96.5)	81 (65.3)	
Female	51 (25.0)	6 (26.1)	2 (3.6)	43 (34.7)	
Mean age (years)	38.8 ± 9.8	40.6 ± 11.5	37.9 ± 9.1	38.9 ± 9.8	0.534
Contract-type					<0.001
CDI	38 (18.6)	9 (39.1)	15 (26.3)	14 (11.3)	
CDD	16 (7.8)	4 (17.4)	8 (14.0)	4 (3.2)	
CDC	150 (73.5)	10 (43.5)	34 (59.6)	106 (85.5)	
Median duration of employment (years)	10.0 ± 7.8	12.0 ± 9.0	10.0 ± 7.3	9.0 ± 7.8	0.257
Socio-economic status					0.005
Low	83 (40.7)	8 (34.8)	18 (31.6)	57 (46.0)	
Middle	103 (50.5)	9 (39.1)	32 (56.1)	62 (50.0)	
High	18 (8.8)	6 (26.1)	7 (12.3)	5 (4.0)	
Exposure to:					
Agrochemicals	48 (23.5)	0 (0.0)	2 (3.5)	46 (37.1)	<0.001
Heavy metals	7 (3.4)	1 (4.3)	6 (10.5)	0 (0.0)	<0.001
Heat	130 (63.7)	0 (0.0)	9 (15.8)	121 (97.6)	<0.001
Alcohol use	167 (81.9)	20 (87.0)	53 (93.0)	96 (77.4)	0.031
Chronic use of NSAIDs	134 (65.7)	13 (56.5)	39 (68.4)	82 (66.1)	0.589
Chronic use of herbal medicines	132 (64.7)	15 (65.2)	41 (71.9)	76 (61.3)	0.379
Family history of CKD	1 (0.5)	0 (0.0)	1 (1.8)	0 (0.0)	0.274
Obesity and overweight	64 (31.4)	11 (47.8)	15 (26.3)	38 (30.6)	0.165
Mean BMI (kg/m^2^)	23.8 ± 3.7	24.6 ± 4.4	23.5 ± 3.1	23.8 ± 3.8	0.516
Tobacco use	31 (15.2)	2 (8.7)	8 (14.0)	21 (16.9)	0.576
Hypertension	20 (9.8)	4 (17.4)	3 (5.3)	13 (10.5)	0.236
Mean SBP^a^ (mmHg)	132.6 ± 15.2	137.1 ± 21.7	128.8 ± 13.4	133.4 ± 14.3	0.052
Mean DBP^a^ (mmHg)	79.6 ± 10.1	83.2 ± 13.5	78.6 ± 9.0	79.4 ± 9.9	0.171
Diabetes	0 (0.0)	0 (0.0)	0 (0.0)	0 (0.0)	–
Mean FBS^a^ (mg/dL)	90.3 ± 13.5	90.5 ± 11.9	94.2 ± 14.3	88.5 ± 13.6	0.458
Mean RBS^a^ (mg/dL)	107.8 ± 20.3	111.5 ± 15.2	102.1 ± 16.5	109.8 ± 22.2	0.084
Mean serum creatinine^a^ (mg/L)	10.9 ± 1.6	10.6 ± 1.3	11.6 ± 1.5	10.6 ± 1.6	<0.001
Mean eGFR^a^ (ml/min)	109.6 ± 20.5	110.3 ± 21.9	107.9 ± 16.5	110.3 ± 21.9	0.760

Conversion factors for units: serum creatinine in mg/dL to μmol/L, × 88.4

*BMI* Body mass index *CDI* Permanent contract of non-specific duration, *CDD* Permanent contract of specific duration, *CDC* Temporary/seasonal contract, n: Absolute frequency, *eGFR* Estimated glomerular filtration rate, *NSAIDs* Non-steroidal anti-inflammatory drugs

^a^Values based on measurements at baseline

As would be expected, field workers were more exposed to agrochemicals (*p* < 0.001) and heat (p < 0.001) compared to the other workspaces whereas factory workers were significantly more exposed to heavy metals (*p* = 0.001) and they consumed more alcohol (*p* = 0.016). The mean body mass index (BMI) was 23.8 kg/m^2^, 23.2 kg/m^2^ in men and 25.6 kg/m^2^ in women. There were no diabetics and <10% of the population had hypertension (Table 1).

### Urine abnormalities

**Fig. 2:**
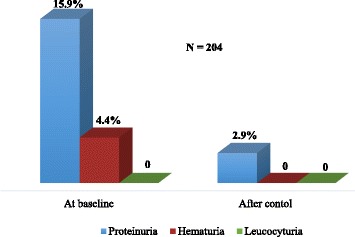
Prevalence of urine abnormalities: The figure shows the change in prevalence of urine abnormalities (proteinuria, hematuria and leucocyturia) over time, from baseline to 3 months later after control tests were done

The most prevalent urine abnormality was proteinuria and its prevalence decreased from 15.9% to 2.9% after the post-interval tests (Fig. 2).

### Prevalence of CKD

In all, 6 out of 204 (2.9%) participants had persistent proteinuria which was mild in every case. One out of 204 (0.5%) participants had persistent eGFR <60 ml/min. The mean serum creatinine was 1.09 ± 0.16 mg/dl (range: 0.78–1.75) while the mean eGFR was 109.63 ± 20.46 (range: 48–168).The overall prevalence of CKD was 3.4% [95% CI = 1.0–6.0]. The factory workers were most affected with a prevalence of 7% compared to the 0% and 2.4% of office and field workers respectively. Chronic use of herbal medicines (*p* < 0.001) and alcohol (*p* < 0.001) was significantly higher in the participants with CKD compared to their counterparts. Even after excluding the participant with an abnormal GFR, the participants with CKD had a lower GFR though the difference was not statistically significant (Table 2).

**Table 2:** Comparison of study population according to CKD status (*N* = 204)

Variable	Total *N* = 204	CKD, *N* = 7 n (%)	Non-CKD, *N* = 197 n (%)	*p*-value
Mean age (years)	38.8 ± 9.8	45.1 ± 8.6	38.6 ± 9.8	0.080
Mean SCr (mg/L)	10.9 ± 1.6	12.6 ± 2.5	10.8 ± 1.5	0.003
Mean eGFR (mg/dl)	109.6 ± 20.5	94.2 ± 26.7	110.2 ± 20.1	0.042
Mean eGFR_2_^a^ (mg/dl)	109.9 ± 20.1	102.0 ± 18.7	110.2 ± 20.1	0.325
Alcohol use	169 (81.9)	7 (100.0)	162 (82.2)	0.606
Chronic use of NSAIDs	134 (65.7)	6 (85.7)	128 (65.0)	0.426
Chronic use of herbal medicines	132 (64.7)	7 (100.0)	125 (63.5)	0.045
Overweight and obesity	64 (31.4)	1 (14.3)	63 (32.0)	0.437
Tobacco use	31 (15.2)	2 (28.6)	29 (14.7)	0.288
Hypertension	20 (9.8)	1 (14.3)	19 (9.7)	0.520
Family history of CKD	1 (0.5)	0 (0.0)	1 (0.5)	0.966

*CKD* Chronic kidney disease, *eGFR* Estimated glomerular filtration rate, *n* Absolute frequency, *NSAIDs* Non-steroidal anti-inflammatory drugs

^a^Comparison of the mean eGFRs excluding the one participant with eGFR <60 ml/min

### Factors associated with CKD

In bivariate analysis (Table 3), age ≥ 40 years (*p* = 0.032) and the use of herbal medicines (*p* = 0.045) were found associated with CKD. With a threshold of *p* ≤ 0.3, we retained age, duration of employment, socioeconomic status, workspace, chronic use of NSAIDs, tobacco use and obesity/overweight as covariates for a binary logistic regression analysis (Table 3); after which, only age ≥ 40 years was independently associated with CKD [OR = 18.7, 95% CI = 1.5–236.4, *p* = 0.024].

**Table 3:** Factors associated with chronic kidney disease

Variable	CKD						
	n (%)	OR	95% CI	*p* value	AOR	95% CI	Adjusted p
Age ≥ 40 years	6 (85.7)	7.5	0.9–60.8	0.032	18.7	1.5–236.4	0.024
Male gender	6 (85.7)	2.0	0.2–16.2	0.442	–	–	–
Middle SES	4 (57.1)	5.9	0.7–48.0	0.062	5.0	0.5–45.7	0.157
Duration of employment ≥15 years	3 (42.9)	2.1	0.5–9.0	0.272	0.5	0.1–3.5	0.442
Factory workspace	4 (57.1)	3.4	0.8–14.9	0.097	–	–	0.998
Seasonal work contract	4 (57.1)	0.5	0.1–2.1	0.384	–	–	–
Exposure to:							
-Agrochemicals	1 (14.3)	0.5	0.1–4.4	0.480	–	–	–
-Heavy metals	0 (0.0)	–	–	0.780	–	–	–
-Heat	4 (57.1)	0.8	0.2–3.3	0.498	–	–	–
Hypertension	1 (14.3)	1.5	0.2–12.1	0.520	–	–	–
Use of herbal medicines	7 (100.0)	–	–	0.045	–	–	–
Alcohol consumption	7 (100.0)	–	–	0.262	–	–	–
Obesity and overweight	1 (14.3)	0.4	0.0–3.0	0.297	0.2	0.0–2.2	0.209
NSAID use	6 (85.7)	3.2	0.4–27.4	0.241	0.4	0.0–3.2	0.350
Tobacco use	2 (28.6)	0.4	0.1–2.3	0.288	0.5	0.1–3.1	0.470

*AOR* Adjusted odds ratio, *CI* Confidence interval, *CKD* Chronic kidney disease, *n* Absolute frequency, *NSAIDs* Non-steroidal anti-inflammatory drugs, *OR* Odds ratio, *SES* Socioeconomic status

## Discussion

The aim of this study was to determine the prevalence and associated factors of CKD in a sugarcane plantation in Cameroon. We found a low CKD prevalence of 3.4% with persistent non-nephrotic range proteinuria being the main marker of kidney damage. Age ≥ 40 years was the only independent predictor of CKD in our study population.

The reported prevalence of CKD in plantation workers ranges from 2.3% [12] to 50.2% [6] depending on the definition of CKD, and the study setting. It is highest when the diagnosis of CKD is based on a single raised urinary protein excretion or serum creatinine, when the workers work for longer hours, are more exposed to heat and when the plantation is situated at a low altitude (≤ 300 masl) [8]. Sugarcane plantation workers in Central America perform similar tasks and use similar agrochemicals to what we observed in our study but for a difference in the altitude of the study settings and the working conditions [7, 8, 13–15]. The study settings at higher altitudes (≥ 500 masl) have temperatures about 4 °C lower than those at lower altitudes [13]. This difference in temperature means that the workers at lower altitudes are more exposed to heat and consequent dehydration which are some of the putative causes of CKD in these workers [4]. Our low prevalence at an altitude of ~516 masl, is consistent with recent findings in El Salvador which showed a prevalence of 4.2% for proteinuria and 2.5% for low eGFR (<60 ml/min/1.73m^2^), in a sugarcane farming community at a similar altitude of ~ 500 masl [8]. However, higher prevalence rates have been reported, especially in settings at lower altitudes. In Nicaragua, Torres et al. reported 10.3% with low eGFR [7] and in another setting at a lower altitude in El Salvador, Peraza et al. reported 12.4% [8]. The difference in altitude between their respective study sites (100 masl and 50 masl) compared to ours may explain this difference in prevalence. Putting our findings into the national context, our prevalence was lower than the 14.2% estimated prevalence in the general community in a rural setting in Cameroon [3]. The difference may be explained partly by the low prevalence rates of the traditional risk factors of CKD: diabetes, hypertension, overweight and obesity in our study population.

Kidney disease in agricultural communities has been observed to be tubulo-interstitial in nature, presenting with little or no proteinuria, and is suspected to occur sub-clinically even though eGFR values are within the normal range. We found a low (2.9%) prevalence of proteinuria in our study population and when present, it was mild, in concordance with various studies irrespective of altitude [8, 14, 15]. We observed that the mean eGFR in the participants with CKD, though within the normal range, was significantly lower than in those without. This is expected, as the calculation of eGFR depends on serum creatinine which is a late marker of kidney damage, only increasing when kidney function has declined by about 50% [16]. Other markers of kidney tubulo-interstitial injury have been identified such as neutrophil gelatinase-associated lipocalin (NGAL) and N-acetyl-beta-D-glucosaminidase (NAG) which may increase earlier in the course of the disease [17, 18]. Assays of these markers in workers in Nicaragua showed an increase over a 6-month harvest period and they were each significantly associated with decrease in eGFR [15]. Control tests to confirm the chronicity of signs of kidney damage is important to prevent overestimation of CKD prevalence. In our study, about 85% of those with urine abnormalities showed none after post-interval tests. These findings imply that proteinuria and other signs of kidney damage are transient or acute, in support of the assertion that CKD in this risk group is due to recurrent episodes of acute kidney damage [19].

We observed that 57% of our participants with CKD worked in the factory. This contrasts with the predilection for field workers observed by Laws et al. in Nicaragua [14]. A difference in the working conditions in both settings may account for this difference. According to an independent report on the working conditions of sugarcane plantation workers in Nicaragua [20], the field workers worked for about 12 h a day, 7 days a week. These working conditions contrast with what we observed where workers typically are in the field for an average of 5 h a day, 6 days a week and usually finish their work around 10 a.m., before the sun is brightest and hottest. However, in our population, the factory workers work about 8 h a day, 6 days a week. Furthermore, the same report purports that workers have access to half the recommended amount of water under these circumstances by the United States Environmental Protection Agency [20]. In our setting, there are several 3000 L potable water tanks in the plantation within a walking distance of every field worker at any time. This ensures that workers have unlimited access to potable water throughout the workday which may be a protective factor for the field workers and may explain the differences observed.

Several factors have been associated with CKD in sugarcane plantation workers, including: older age, field work, working in lower altitudes, exposure to agrochemicals, increased duration of employment and chronic use of NSAIDs. Though chronic use of NSAIDs and herbal medicines were frequent potential risk factors irrespective of workspace, we found only age ≥ 40 years to be an independent predictor of CKD. This is consistent with findings in El Salvador where age > 40 years was a predictor of CKD [8]. We found no association between exposure to agrochemicals and CKD. This corroborates with the observation of Laws et al. in Nicaragua, who evaluated changes in eGFR over a 6-month period, and found that agrochemical applicators (those who are most directly in contact with agrochemicals) showed the least decline in eGFR compared to the workers in other job categories in the field [14]. This suggests that exposure to agrochemicals may not be a cause of CKD in this risk group. However, several studies [21–23] have shown a possible link of agrochemicals to CKD and hence it is a factor which needs to be studied more extensively. Even though, herbal medicines, alcohol and NSAIDs have been demonstrate as risk factors for CKD in similar settings [3, 24], we found no significant association between these factors and CKD in our study population. Yet other factors are suspected to be potential risk factors for CKD in similar populations, including leptospirosis infection, genetic factors, exposure to metals in volcanic soils etc. Though we did not assess them in our study, we believe they may play a role and hence should be further investigated.

### Limitations and strengths

One of the limitations of our study was the small size of our study population. However, the representative random stratified sampling method we used, and our number of participants which exceeded the calculated minimum sample size, reduces sampling bias and hence permits the inference of our findings to the general working population in SOSUCAM. Furthermore, studies carried out in other countries like Nicaragua (*N* = 284) [14] and El Salvador (*N* = 224) [6] had similar sample sizes which assisted comparability of our findings. Furthermore, that we carried out our study in only one of the two sugarcane plantations in Cameroon potentially limits our work. However, the other plantation in Nkoteng (also part of SOSUCAM), which is at an even higher altitude (~580 masl), performs similar practices with work conditions as described in the Mbandjock plant. Therefore, we believe our results can be generalised to reflect the situation of all sugarcane plantation workers. Despite these, our study is the first in Cameroon to describe the burden of CKD in sugarcane plantation workers and among the few reported in SSA. This therefore constitutes a significant contribution to the literature on CKD in Africa.

## Conclusion

This study depicts a low prevalence of CKD among sugarcane plantation workers in Cameroon. Also, plantation workers aged 40 years and beyond are more likely to have CKD. Furthermore, our study accentuates the need for a control urine test in the evaluation of CKD prevalence in observational studies. Dehydration and heat stress being one of the putative risk factors for CKD in similar populations, we believe the practices in the company which serve to reduce their effects may play a role in protecting the workers from CKD. We recommend similar studies with larger sample sizes to be conducted in other plantations and other sub-Saharan countries so as to illuminate the true burden of CKD in this part of the world, as well as to identify the causative factors of CKD in sugarcane workers in particular.

## Abbreviations

ANOVA: Analysis of variance
BMI: Body mass index
CKD: Chronic kidney disease
CKD-EPI: Chronic kidney disease epidemiology collaboration
CVD: Cardiovascular disease
eGFR: Estimated glomerular filtration rate
ESRD: End-stage renal disease
IQR: Interquartile range
Masl: Meters above sea level
NAG: N-acetyl-beta-D-glucosaminidase
NECHHR: National ethics committee for human health research
NGAL: Neutrophil gelatinase-associated lipocalin
NSAIDs: Non-steroidal anti-inflammatory drugs
SD: Standard deviation
SOSUCAM: Cameroon sugar company
SPSS: Statistical package for social sciences
SSA: Sub-Saharan Africa
WHO: World Health Organization
